# Supplementary figures and images for: Improved DOP-PCR (iDOP-PCR): A robust and simple WGA method for efficient amplification of low copy number genomic DNA
Source: PLoS One. 2017 Sep 11;12(9):e0184507. doi: 10.1371/journal.pone.0184507 (PMC5593185; doi:10.1371/journal.pone.0184507)

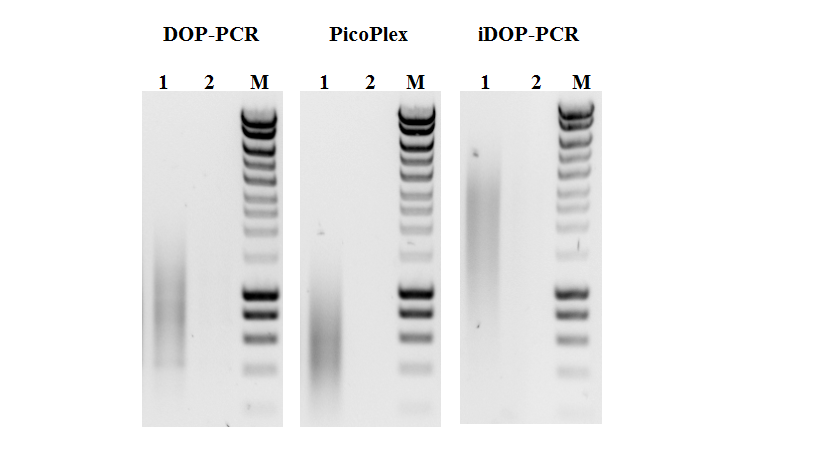

Supplement: S1 Fig — The libraries were obtained by DOP-PCR, PicoPlex and iDOP-PCR methods from 15 pg (Lanes 1) and 0 pg (negative controls, Lanes 2) of the input human gDNA. M– 1 kb DNA Ladder. (TIF) [file pone.0184507.s001.tif]
