# Supplementary material for: Improved DOP-PCR (iDOP-PCR): A robust and simple WGA method for efficient amplification of low copy number genomic DNA
Source: PLoS One. 2017 Sep 11;12(9):e0184507. doi: 10.1371/journal.pone.0184507 (PMC5593185; doi:10.1371/journal.pone.0184507)
Supplement: S1 Table — Data for each point were obtained from analisis of 6 WGA samples. (DOC) [file pone.0184507.s002.doc]

**S1 Table. Average yield of DNA amplified by DOP-PCR, PicoPlex and iDOP-PCR.**

| **Amount of gDNA template for WGA** | **DOP-PCR** | **PicoPlex** | **iDOP-PCR** |
| --- | --- | --- | --- |
| 15 000 pg | 46 ng/μl | 50 ng/μl | 42 ng/μl |
| 1 500 pg | 47 ng/μl | 47 ng/μl | 39 ng/μl |
| 150 pg | 50 ng/μl | 46 ng/μl | 45 ng/μl |
| 15 pg | 40 ng/μl | 43 ng/μl | 48 ng/μl |
| 0 pg  (negative control) | 6 ng/μl | 3 ng/μl | 2 ng/μl |

Data for each point were obtained from analisis of 6 WGA samples.
