# Supplementary material for: Improved DOP-PCR (iDOP-PCR): A robust and simple WGA method for efficient amplification of low copy number genomic DNA
Source: PLoS One. 2017 Sep 11;12(9):e0184507. doi: 10.1371/journal.pone.0184507 (PMC5593185; doi:10.1371/journal.pone.0184507)
Supplement: S2 Table — In each wgaDNA and non-amplified gDNA sample, 38 allels were analyzed. Statistic data for each starting amount of gDNA amplified by each WGA method were obtained from the assay of six separate wgaDNA samples. Total N = 6 x 38 allels = 228 allels (100%). Allele concordance was calculated as a percentage of concordant alleles (Table A in S2 Table). Allele drop out (ADO) was calculated as a percentage of dropping-out concordant alleles (Table B in S2 Table). Allele drop in (ADI) was calculated as a percentage of dropping-in discordant alleles (Table C in S2 Table). (DOC) [file pone.0184507.s003.doc]

**S2 Table. Multiplex STR genotyping of WGA samples and non-amplified gDNA.**

In each wgaDNA and non-amplified gDNA sample, 38 allels were analyzed. Statistic data for each starting amount of gDNA amplified by each WGA method were obtained from the assay of six separate wgaDNA samples. Total **N = 6 x 38 allels = 228 allels (100%).**

**(A) Allele concordance** was calculated as a percentage of concordant alleles.

**(B)** **Allele drop out (ADO)** was calculated as a percentage of dropping-out concordant alleles.

**(C) Allele drop in (ADI)** was calculated as a percentage of dropping-in discordant alleles.

**(A) Allele concordance**

| **WGA method** | **gDNA template for WGA (pg)** | **N**  **number of alleles (total/per sample)** | **Number of concordant alleles** | | | | | | | | **Standard deviation (SD)** | **Concordance (%)** |
| --- | --- | --- | --- | --- | --- | --- | --- | --- | --- | --- | --- | --- |
|  |  |  | **Sample 1** | **Sample 2** | **Sample 3** | **Sample 4** | **Sample 5** | **Sample 6** | **Total (Σ)** | **Mean per sample** |  |  |
| Non-amplified gDNA | - | 228/38 | 38 | 38 | 38 | 38 | 38 | 38 | 228 | 38 | - | 100% |
| PicoPlex | 15 000  1 500  150  15 | 228/38 | 28  23  20  18 | 27  24  18  17 | 29  23  19  18 | 28  21  20  15 | 25  20  18  18 | 29  24  21  14 | 166  135  116  100 | 27.7  22.5  19.3  16.7 | 1.506  1.643  1.211  1.751 | 72.8%  59.2%  50.9%  43.9% |
| iDOP-PCR | 15 000  1 500  150  15 | 228/38 | 34  32  29  23 | 32  33  29  19 | 32  33  29  20 | 31  30  30  21 | 33  29  30  19 | 30  33  32  21 | 192  190  179  123 | 32.0  31.7  29.8  20.5 | 1.414  1.751  1.169  1.517 | 84.2%  83.3%  78.5%  53.9% |
| DOP-PCR | 15 000  1 500  150  15 | 228/38 | 8  0  0  1 | 6  4  0  0 | 7  2  1  0 | 13  0  1  0 | 8  2  1  1 | 12  2  1  1 | 54  10  4  3 | 9.0  1.7  0.7  0.5 | 2.828  1.506  0.516  0.548 | 23.7%  4.4%  1.8%  1.3% |

**(B)** **Allele drop out (ADO)**

| **WGA method** | **gDNA template for WGA (pg)** | **N**  **number of alleles (total/per sample)** | **Number of dropping-out concordant alleles** | | | | | | | | **Standard deviation (SD)** | | **Total ADO errors (%)** | | |
| --- | --- | --- | --- | --- | --- | --- | --- | --- | --- | --- | --- | --- | --- | --- | --- |
|  |  |  | **Sample 1** | **Sample 2** | **Sample 3** | **Sample 4** | **Sample 5** | **Sample 6** | **Total (Σ)** | **Mean per sample** | |  | | |  |
| Non-amplified gDNA | - | 228/38 | 0 | 0 | 0 | 0 | 0 | 0 | 0 | 0 | | - | | 0% | |
| PicoPlex | 15 000  1 500  150  15 | 228/38 | 10  15  18  20 | 11  14  20  21 | 9  15  19  20 | 10  17  18  23 | 13  18  20  20 | 9  14  17  24 | 62  93  112  128 | 10.3  15.5  18.7  21.3 | | 1.505  1.643  1.211  1.751 | | 27.2%  40.8%  49.1%  56.1% | |
| iDOP-PCR | 15 000  1 500  150  15 | 228/38 | 4  6  9  15 | 6  5  9  19 | 6  5  9  18 | 7  8  8  17 | 5  9  8  19 | 8  5  6  17 | 36  38  49  105 | 6.0  6.3  8.2  17.5 | | 1.414  1.751  1.169  1.516 | | 15.8%  16.7%  21.5%  46.1% | |
| DOP-PCR | 15 000  1 500  150  15 | 228/38 | 30  38  39  37 | 32  34  38  38 | 31  36  37  39 | 25  38  36  37 | 30  36  37  37 | 26  36  37  37 | 174  218  224  225 | 29.0  36.3  37.3  37.5 | | 2.828  1.506  1.033  0.837 | | 76.3%  95.6%  98.2%  98.7% | |

**(C) Allele drop in (ADI)**

| **WGA method** | **gDNA template for WGA (pg)** | **N**  **number of alleles (total/per sample)** | **Number of dropping-in discordant alleles** | | | | | | | | **Standard deviation (SD)** | | **Total ADI errors (%)** | | |
| --- | --- | --- | --- | --- | --- | --- | --- | --- | --- | --- | --- | --- | --- | --- | --- |
|  |  |  | **Sample 1** | **Sample 2** | **Sample 3** | **Sample 4** | **Sample 5** | **Sample 6** | **Total (Σ)** | **Mean per sample** | |  | | |  |
| Non-amplified gDNA | - | 228/38 | 0 | 0 | 0 | 0 | 0 | 0 | 0 | 0 | | - | | 0% | |
| PicoPlex | 15 000  1 500  150  15 | 228/38 | 1  1  0  2 | 2  1  1  1 | 0  1  1  1 | 0  0  2  3 | 2  1  1  1 | 2  1  0  1 | 7  5  5  9 | 1.2  0.8  0.8  1.5 | | 0.983  0.408  0.753  0.837 | | 3.0%  2.2%  2.2%  3.9% | |
| iDOP-PCR | 15 000  1 500  150  15 | 228/38 | 5  4  3  3 | 3  5  4  1 | 5  5  4  1 | 5  4  3  1 | 4  3  4  1 | 3  4  3  2 | 25  25  21  9 | 4,2  4,2  3.7  1.5 | | 0.983  0.753  0.816  0.837 | | 10.9%  10.9%  9.2%  3.9% | |
| DOP-PCR | 15 000  1 500  150  15 | 228/38 | 0  1  0  0 | 0  1  0  0 | 0  0  0  0 | 1  0  0  0 | 0  0  0  0 | 0  0  0  0 | 1  2  0  0 | 0.2  0.3  0  0 | | 0.374  0.516  -  - | | 0%  0.9%  0%  0% | |
